# Optimizing nitrogen application position to change root distribution in soil and regulate maize growth and yield formation in a wide–narrow row cropping system: pot and field experiments

**DOI:** 10.3389/fpls.2024.1298249

**Published:** 2024-01-24

**Authors:** Shiyong Zhou, Pan Xia, Junping Chen, Qijiao Xiong, Guanhan Li, Jingyi Tian, Bozhi Wu, Feng Zhou

**Affiliations:** Faculty of Agronomy and Biotechnology, Yunnan Agricultural University, Kunming, Yunnan, China

**Keywords:** wide-and narrow-row cropping system, maize, yield, foraging behavior, nitrogen

## Abstract

The wide-and narrow-row cropping technology used for maize has the advantages of protecting cultivated soil and improving the population structure in maize fields. However, the relationship between nitrogen application position and root interactions has not been determined. Through pot and field experiments, we evaluated the effects of two nitrogen application positions ((narrow row nitrogen application (RC) and wide row nitrogen application (RN)) and two nitrogen application regimens ((high nitrogen(HN) and low nitrogen(LN)) on root growth and yield composition of wide-narrow row maize during the flowering and harvest stages. In field experiments, RC increased the biomass, length and surface area of competing roots (narrow-row roots, CR) at the flowering stage. The yield and agronomic efficiency of N(AEN) and partial factor productivity of N(PFPN) were increased by RN compared to RC under HN, However, the AEN under LN was significantly lower; There was no significant effect on maize growth and biomass allocation at the same level of application of N. At the flowering stage, the results of CR and non-competing roots (wide-row roots, NCR) was consistent under pot experiments and the field experiments, and the yield under RN was also higher than that under RC, although the difference was not significant. Furthermore, according to the principal component analysis and correlation analysis, the competing roots were the main factor influencing yield and AEN. In conclusion, our study showed that RN is a useful fertilization method to improve overall productivity. All in all, how roots coordinate neighbors and nitrogen spatial heterogeneity is a complex ecological process, and its trophic behavior deserves further study.

## 1 Introduction

The achievement of efficient nutrient utilization, the improvement of crop productivity, and the reduction of competition among individuals in the population are the primary goals of optimizing fertilization methods, as well as the requirements of achieving intensive agricultural development (Liu et al., 2022; Wu et al., 2022a). Strip fertilizer has been widely discussed as a method for improving fertilizer utilization efficiency in modern agricultural production. Strip tillage and deep strip application of nitrogen have been reported to significantly improve maize growth, nutrient absorption, and grain yield (Nash et al., 2013; Jia et al., 2018). It has also been reported that applying a single nutrient belt or a combined nutrient belt near the maize planting ditch at planting can improve nutrient absorption and utilization efficiency in the early stage and promote plant growth under humid soil conditions (Rutan and Steinke, 2018; Quinn et al., 2020). This is not only due to the heterogeneity of soil nutrients created by strip fertilization, which leads to local nutrient concentrations, stimulates lateral root development, and establishes an ideal root configuration, thus increasing nutrient absorption and crop yield, but also due to the concentration of soil nutrient resources by strip fertilization, which regulates root morphology and physiology to respond to changes in environmental conditions, enhancing the ability of the plant to obtain resources. In addition, strip fertilization can also reduce ammonia volatilization in the soil, reduce the fixation and adsorption of phosphorus and potassium by soil particles, and improve the effectiveness of phosphorus and potassium. For example, in the no-tillage mode, the application of urea strips significantly reduces ammonia volatilization by 52% compared to spraying (Trapeznikov et al., 2003; Rochette et al., 2009).

The depth of strip fertilization is also an important factor that affects fertilizer of efficiency. Many studies have shown that increasing the depth of fertilization to an appropriate level can increase the nitrogen content of nitrate in the soil, improve the photosynthetic characteristics of the leaves, increase the rate of fertilizer utilization, and indirectly improve the nutritional status of maize plants (Aluoch et al., 2022; Wu et al., 2022b). For example, the seed yield, oil yield, and fertilizer use efficiency of rapeseed fertilized at a depth of 10 cm were significantly greater than those at 5 and 15 cm (Chen et al., 2023). In the maize–wheat rotation system, a strip fertilization depth of 15 cm can effectively increase the root length density, root surface area density, nitrogen absorption, leaf area index, dry matter accumulation, and radiation interception ability of maize and, at the same time, increase the yield of subsequent crops (wheat) (Chen et al., 2022). In monoculture maize, compared to conventional nitrogen application, increasing the fertilization depth to 12 cm and reducing the amount of slow-release fertilizer applied by 20% still maintained a higher maize yield (Hu et al., 2023). In summary, although strip fertilization is superior to conventional hole fertilization and scatter fertilization in fertilizer utilization rate and yield, the above research does not consider the influence of the interaction between crop roots under intensive planting conditions. In the wide-and narrow-rows of the maize planting systems, the roots between the narrow-rows are highly overlapped, which has a competitive effect on nutrients. We changed the position of strip application (fertilization between narrow-rows: fertilizer and roots interaction; wide interrow fertilization: root communication but no fertilizer effect) to change the complex communication between crop roots and to explore how crop roots respond to the strip application position in an intensive planting system according to the interaction between roots and fertilizers.

We hypothesized that changing the fertilization position (nitrogen application in overlapping areas or nonoverlapping areas) could change the heterogeneity of soil nitrogen distribution and then change the distribution of inter-plant roots, reduce the competition of inter-plant roots for nutrients in the population, and improve the spatial matching degree between maize roots and soil nutrients, and improve the utilization rate of nutrient resources and crop productivity to realize sustainable agricultural development. Here, a pot experiment using the ‘root splitting’ method and a field validation experiment were used to evaluate the strip application of nitrogen fertilizer in different positions. The purpose of this study was: (1) to determine the effects of nitrogen application at different locations on the spatial distribution and growth of maize roots; (2) to determine the effects of nitrogen fertilizer application at different positions on root cultivation behavior and fertilizer utilization rate; (3) to study the effects of nitrogen fertilization on maize growth, biomass distribution, and yield. Through this study, we hope to better understand the effects of nitrogen application at different locations on root distribution and growth, as well as its effects on crop nutrient utilization and yield. This approach will help provide sustainable agricultural development solutions, promote soil health, and ensure the sustainability of crop production.

## 2 Materials and methods

### 2.1 Field experiment

#### 2.1.1 Experiment site

This experiment was carried out at the Daheqiao Experimental Station (103°16′41”E, 25°31′07”N), Xundian County, Yunnan Agricultural University, from April to August 2023. The altitude of this area is 1850 m, and precipitation is concentrated from June to September. The average annual precipitation is 1180 mm, and the average annual evaporation is 2384 mm. This area belongs to the north subtropical monsoon climate, with an annual average temperature of 15.3 C. The soil was yellow brown soil (GB/T 17296-2009, 17% sand (0.05–2 mm), 38% silt (0.002-0.05 mm), and 45% clay (< 0.002 mm)). The pH of the 0–20 cm soil layer was 6.92, and the contents of total nitrogen, phosphorus, and potassium contents were 1.16, 0.79, and 11.82 g kg^−1^, respectively. The available N, P, and K were 45.26, 20.34, and 83.62 mg kg^−1^, respectively, and the content of organic matter in the soil was 16.88 g kg^−1^.

#### 2.2.2 Experimental design

**Figure 1:**
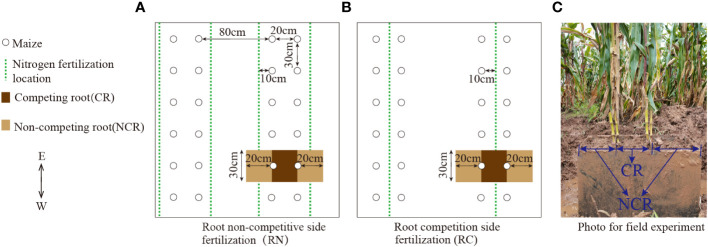
Planting diagram of the field experiment and sampling diagram of the roots. **(A)** Root non-competitive side fertilization (RN). **(B)** Root competition side fertilization (RC). **(C)** Photo of the field experiment. CR, Competing root; NCR, Non-competing root.

There were two experimental factors: nitrogen position and nitrogen level. The nitrogen application position included competitive root side nitrogen application(RC) (i.e., narrow-row side nitrogen application, as shown in **Figure 1B**) and non-competitive root side nitrogen application (RN) (i.e., wide-row side nitrogen application, as shown in **Figure 1A**). The nitrogen level was established with two nitrogen levels: high nitrogen (HN, 9 g/plant) and low nitrogen (LN, 4.5 g/plant). There were four treatments in total, namely a narrow-row with high nitrogen (RCHN), narrow row with low nitrogen (RCLN), wide row with high nitrogen (RNHN), and wide row with low nitrogen (RNLN), which were arranged in a randomized block design. Each treatment was repeated three times, with a plot area of 5 m × 7 m = 35 m^2^ and an interval of 1 m between the plots. Planting specifications were based on wide-and narrow-rows, with wide-rows of 80 cm, narrow- rows of 20 cm, and a plant spacing of 30 cm. The treatments are shown in **Figures 1A, B**, and the plant density was 66,667 plants/ha. The maize was sown on April 3, 2023. Before sowing, 150 kg ha^−1^ calcium superphosphate (P_2_O_5_ < 16%) and 100 kg ha^−1^ potassium sulfate (K_2_O < 52%) were applied uniformly to the 20 cm soil layer at one time. N fertilizer (Urea, N = 46%) was applied in 50%:50% furrow strips at the seedling stage (April 28, 2020) and the booting stage (May 20, 2023), with a depth of 10 cm. Experimental management was carried out as necessary for artificial weeding and pest control.

### 2.2 Pot experiment

#### 2.2.1 Experimental design

**Figure 2:**
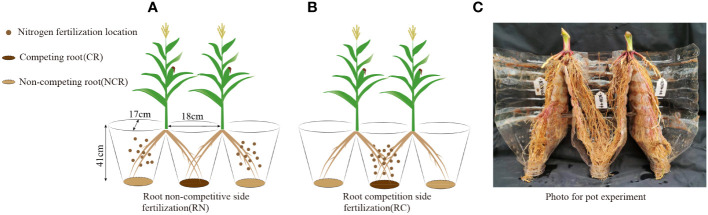
**(A)** Root non-competitive side fertilization (RN), **(B)** Root competition side fertilization (RC), **(C)** Photo of the pot experiment. CR, Competing root; NCR, Non-competing root.

The pot experiment was conducted in a greenhouse at the same position as the field experiment, treatments were the same as in the field experiment: location of nitrogen (no competitive side nitrogen application (**Figure 2A**) and competitive root side nitrogen application (**Figure 2B**) and nitrogen level (high nitrogen and low nitrogen). The tested soils were taken from the field experiment. In this experiment, the ‘root splitting’ technology was used to explore the feeding behavior of the maize roots with different nitrogen application positions, which was an effective way to separate underground parts to prevent the diffusion of soil nutrients between nutrient pots (Yu et al., 2013; in ‘t Zandt et al., 2015). we bundled three plastic bottles with a size of 18 cm × 17 cm × 41 cm (length × width × height) together, put a layer of nylon mesh (0.1 mm aperture) on the bottom to prevent the roots from sticking and to facilitate water penetration, and then cut off a groove with a height of 5 cm from the upper joint to facilitate seedling transplantation. Specific test devices are shown in **Figures 2A, B**. The completely random arrangement was repeated in the greenhouse to prevent uneven illumination. In three conjoined containers, 2.5 g of superphosphate (P_2_O_5_ < 16%) and 4 g of potassium sulfate (K_2_O < 52%) were mixed with sifted dry soil as base fertilizer and applied uniformly in three conjoined containers (approximately 10 kg per containers) at one time. The nitrogen application rate was the same as that in the field experiment, and was applied at 50%:50% of the total fertilizer amount two weeks after maize transplant and at the big bell mouth stage. The weeds were manually removed from the nutrient pots during growth.

#### 2.2.2 Seedling preparation

The seeds of ‘Qiaodan-6’ of the same size and plumpness were soaked in warm water at 45°C for 24 hours and then placed on a wet filter paper seedbed to germinate. After the roots grew, they were cut with sterilized scissors, and the seeds were placed in a nutrition cup (8 cm × 10 cm, cylindrical) to germinate. Seven days after the seeds germinated in the nutrition cup, seedlings of the same size were selected for transplantation on March 20, 2023. After planting, seedlings that died or suffered serious diseases within one week were replaced with new plants, and plants were watered every 5 days to control the intensity of watering to ensure that no leakage occurred at the bottom.

### 2.3 Sampling and observation

In the field experiment, 12 plants were selected for each treatment at the flowering stage (July 6, 2023) and harvest stage (August 20, 2023). For the pot experiment, 16 plants were selected for each treatment at the flowering stage (June 20, 2023) and harvest stage (August 1, 2023), respectively. The leaf area was measured by the length–width coefficient method (maximum leaf length × maximum leaf width × 0.75) and then divided into vegetative and reproductive organs (mature stage). After the enzymes were deactivated at 105°C for 30 min and drying at 70°C until constant weight, the weight of dry matter was measured. In the mature stage under the field experiment, two rows of maize were selected in the middle of each plot for harvesting and yield measurement, and the yield was calculated using a 14% standard water content, which was converted into yield per unit area according to the measured yield area.

Root sampling: In the field experiment, because the roots of two adjacent maize plants between narrow-rows were interlaced with each other, it was impossible to distinguish a complete single root system between narrow-rows, so we considered two plants as a sampling unit. Before sampling, two maize roots were cut into two-halves from the center of the stem along the planting row with two self-made knives (thin steel plate with a thickness of 15 mm, cut to a length of 29 cm, and a width of 40 cm, and one side was polished to make it sharp for root cutting), and then the steel plate (with a thickness of 15 mm) was welded into a cuboid with a length, width, and height of 60 × 30 × 40 cm. The two maize roots were divided into three parts by hammering them into the soil, as shown in **Figure 1C**, and the surrounding soil was digged out. A steel plate with a length of 60 cm and a width of 30 cm was hammered into the bottom of the cuboid to remove the whole cuboid clod, and a nylon mesh cloth was placed on the bottom, and the roots were then brought back to the laboratory for further cleaning, which included the middle competitive roots and the noncompetitive roots on both sides. After being scanned with a root scanner (Shanghai Zhongjing Technology Co., Ltd., China, Shanghai, China, ScanMaker i800 Plus) at the flowering stage, root length, root surface area, and average root diameter were analyzed by WinRHIZO 2019b (Regent Instruments Canada lnc., Quebec City, QB, Canada) and then dried to a constant weight at 70°C, which was recorded as dry matter.

Root sampling in the pot experiment was performed after withholding water for one week. When sampling, the nutrient pot was tapped to scatter the soil, and a cut was made around the nutrient pot at the original position during the maize flowering and harvest periods. They were then rinsed under flowing water, and all collected roots were brought back to the laboratory for further cleaning. After cleaning, the roots were divided into middle pot roots and two pot roots, which were recorded as competitive roots (RC) and noncompetitive roots (NCR), respectively (**Figures 2A, B**). Root scanning was carried out at the flowering stage (same as the field experiment) and then dried to a constant weight at 70°C, which was recorded as the dry matter of the root system. This sampling method maintained the integrity of the root system and allowed comparative analysis of competitive roots and noncompetitive roots.

### 2.4 Data analysis

To clarify the effect of plant resource acquisition ability and allocation strategy, we used the root: shoot ratio to evaluate the competitiveness of plants under the nitrogen application position and nitrogen level and the biomass investment ratio of roots and shoots. In the analysis of the accuracy of roots, we used the ratio of root biomass between high nitrogen patches and low nitrogen patches to measure the adaptive response of plants to heterogeneous nutrients; the higher the ratio, the higher the accuracy of root cultivation (Wu et al., 2012). In this study, CR/NCR biomass under narrow row nitrogen application, and NCR/CR biomass was used under wide row nitrogen application. Moreover, agronomic nitrogen efficiency (AEN) and partial nitrogen productivity (PFPN) were used to evaluate nitrogen efficiency, and the calculation formula was as follows: AEN (kg kg^−1^) = (maize grain yield in the nitrogen application area − maize grain yield in the non-nitrogen application area)/nitrogen application rate; PFPN (kg kg^−1^) = grain yield/nitrogen application rate in the nitrogen application area (Zhang et al., 2020; Liang et al., 2022).

Using SPSS 24.0 as a fixed factor, the yield, harvest index, total root biomass, total root surface area, average root diameter, root:shoot ratio, and nitrogen use efficiency of maize in the field and pot experiments were analyzed by two-factor variance analysis with nitrogen application position and nitrogen level as fixed factors. After analysis, multiple comparisons were made using the least significant difference (LSD) method. The biomass, surface area, length, and average diameter of competitive and noncompetitive roots in field and pot experiments were analyzed using Student’s t-test. Based on PCA and correlation analysis (data were processed standardized with a double-tailed test), the key factors influencing competitive and non-competitive roots on the yield and agronomic efficiency of nitrogen fertilizer were explored. Significance was considered as *P* < 0.05.

## 3 Results

### 3.1 Field experiment

#### 3.1.1 Yield, biomass allocation, and agronomic characteristics

**Figure 3:**
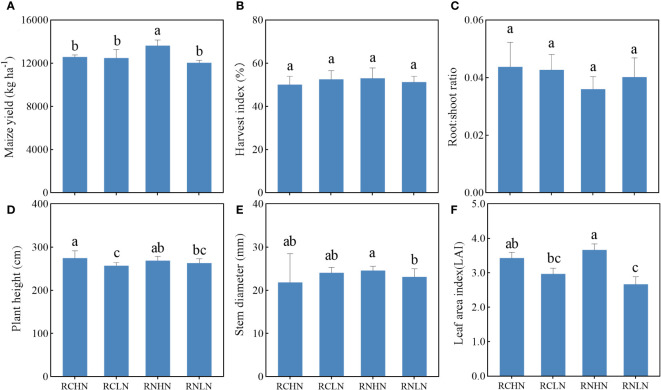
Effects of nitrogen application location and nitrogen level on yield, biomass allocation and agronomic traits in the field experiment. **(A)** Yield. **(B)** Harvest index. **(C)** Root : shoot ratio. **(D)** Plant height. **(E)** Stem diameter and **(F)** Leaf area index. Different lowercase letters indicate a significant difference between treatments (*P* < 0.05).

Under high nitrogen conditions, the maize yield in the RN treatment was significantly higher than that of the RC treatment (*F* = 5.45, *P* = 0.02), while the difference in yield was not significant under low nitrogen conditions. However, RC was higher than RN, and the yield increased by 3.63% (**Figure 3A**), indicating that the wide-row application of high nitrogen had obvious advantages in increasing the yield, while low nitrogen had slight disadvantages. There were no significant differences in the harvest index between treatments (*F* = 1.39, *P* = 0.25), showing the trend: RNHN > RCLN > RNLN > RCLN (**Figure 3B**). Although there was no significant statistical difference between treatments (*F* = 1.72, *P* = 0.19), the root: shoot ratio was higher for RC than for RN (**Figure 3C**), indicating that the RC treatments had greater investment in the root system, which was not conducive to growth and an increase in the yield of the aboveground parts. Under RN, the root:shoot ratio was lower under high nitrogen conditions than under low nitrogen conditions, indicating that an adequate nitrogen fertilizer supply could reduce the root:shoot ratio, which reduces the distribution of dry matter to the underground roots.

The leaf area index (LAI) reflects the total area of the plant leaves, which is closely related to the photosynthesis ability of the plants. Under RC, there were no significant differences between high and low nitrogen levels. Under RN, the LAI of high nitrogen was significantly greater than that of low nitrogen (*F* = 6.13, *P* < 0.01), indicating that treatment with RN could better regulate leaf growth (**Figure 3F**). A higher plant height and stem diameter of crops can help them occupy a higher position in the population, better competition for light and other resources needed for growth, and improve their competitiveness. As shown in **Figures 3D, E**, in RC, the plant height was significantly higher under high nitrogen conditions than under low nitrogen conditions (*F* = 5.24, *P* < 0.01). There was no significant difference in the stem diameter (*F* = 0.95, *P* = 0.33), but under wide-rows, the opposite was true for nitrogen application. These results indicate that the nitrogen application rate significantly affected the leaf area index, plant height, and stem diameter of crops and that a higher nitrogen application rate could achieve greater production potential.

#### 3.1.2 Root growth and cultivation behavior

**Figure 4:**
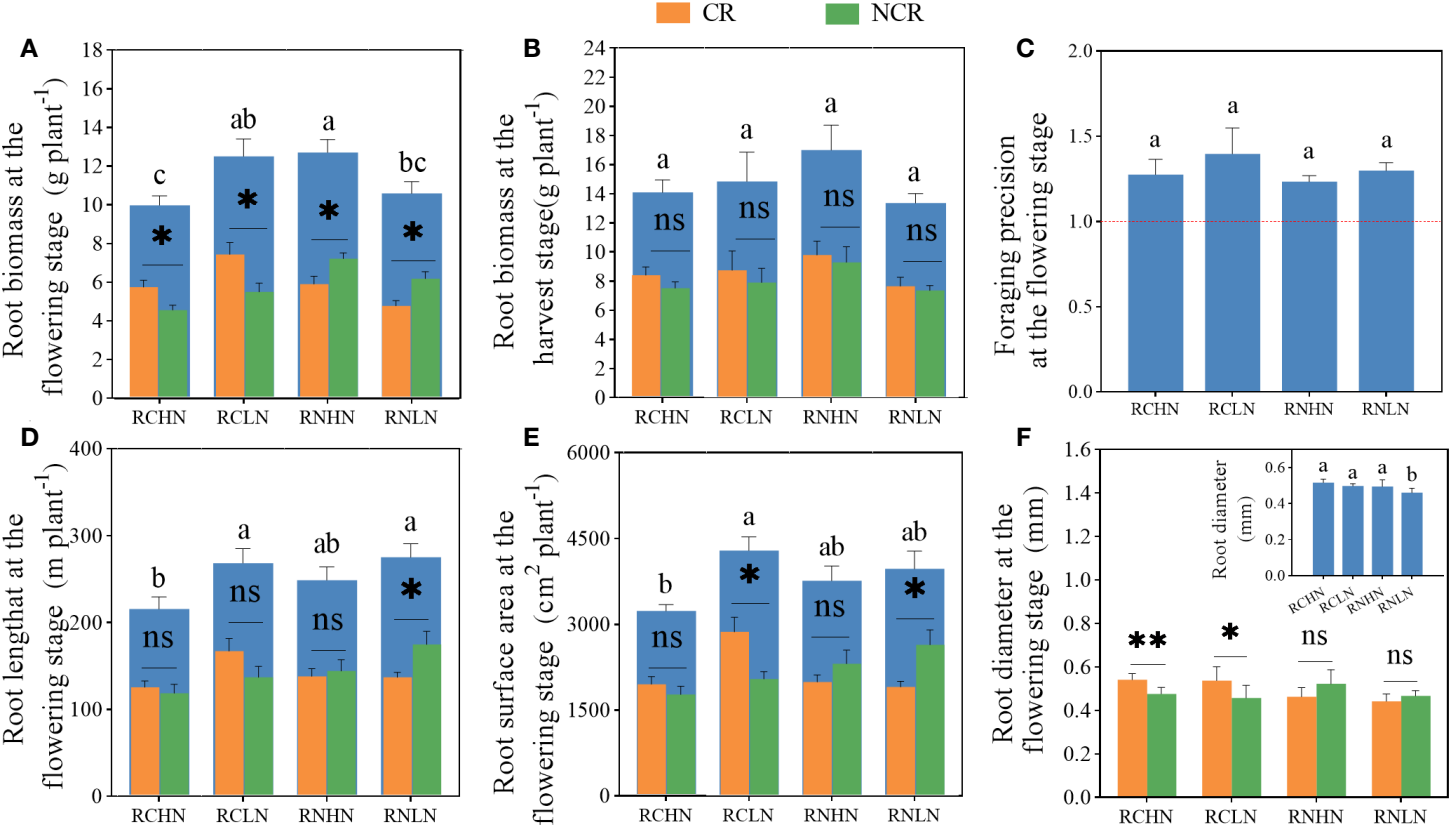
Effects of nitrogen application location and nitrogen level on root traits and foraging precision in the field experiment. **(A)** Root biomass at the flowering stage. **(B)** Root biomass at the harvest stage. **(C)** Foraging precision at the flowering stage. **(D)** Root length at the flowering stage. **(E)** Root surface area at the flowering stage. **(F)** Root diameter at the flowering stage; CR, Competitive root; NCR, Non-competiting root. Different lowercase letters indicate significant differences between treatments (P< 0.05). ns,* and ** indicate significance between the CR and NCR (P>0.05, P<0.05, and P<0.01), respectively.

For total root biomass, RCLN was significantly higher than RCHN at the flowering stage (**Figure 4A**), while RN was the opposite (*F* = 4.02, *P* = 0.02). Moreover,CR biomass was significantly higher than that of NCR biomass in RC (high nitrogen: *F* = 0. 35, *P* = 0. 02; low nitrogen: *F* = 0.52, *P* = 0.03). The biomass of CR was significantly less than that of NCR biomass in RN (high nitrogen: *F* = 0.70, *P* = 0.02, low nitrogen: *F* = 1.27, *P* = 0.001). At maturity, there was no significant difference neither in total biomass of CR nor NCR biomass between treatments, but in the trend of total biomass, CR and NCR biomass was consistent during the flowering period for narrow row nitrogen application. However, in wide-row nitrogen application, the CR and NCR biomass exhibited opposite trends during the flowering period (**Figure 4B**), indicating that after the flowering period, the field nitrogen was exhausted and that the root system grew toward the roots of neighboring plants to absorb nutrients. Moreover, there was no significant difference in the foraging precision (*F* = 0.55,*P* = 0.65) (**Figure 4C**), which were greater than 1, indicated that the greater degree to which the plant was heterogeneous in terms of root adaptation to nitrogen, the greater degree to which the plant was seeking culture.

The root length and root surface area exhibited the same trend (**Figures 4D, E**), showing that under the narrow-row nitrogen application, these values were significantly greater under low nitrogen application than under high nitrogen application (root length: *F* = 5.25, *P* = 0.008; surface area: *F* = 3.40, *P* = 0.038). In the wide-row nitrogen application, there was no significant difference between the high nitrogen and low nitrogen application. Compared with the root length and surface area of CR and NCR, only was NCR significantly higher than CR under the application of wide-row nitrogen with low nitrogen (root length: *F* = 3.34, *P* = 0.045; surface area: *F* = 3.60, *P* = 0.039). When comparing the root length and surface area ratios of different diameters, the total root length ratio of high nitrogen application was higher than that of low nitrogen application, and the root length of RCHN, RCLN, RNHN, and RNLN with diameters less than 2.5 mm accounted for 96.45, 97.59, 97.67 and 97.95%, respectively, of the total root length, which was 81.09, 82.26, 81.92 and 83.96% of the total surface area. The length and surface area of roots larger than 4 mm in diameter under RNHN treatment were significantly higher than those in the RNLN treatment, indicating that the roots were sensitive to nitrogen under low nitrogen application (**Supplementary Tables 1**, **2**). Root diameter is closely related to the ability of plants to absorb water and nutrients. The application of high nitrogen was significantly higher than the application of low nitrogen when nitrogen was applied in wide-rows (*F* = 5.08, *P* = 0.009), but there was no significant difference when nitrogen was applied in narrow-rows. Specifically, the diameter of the competitive roots was significantly higher than that of non-competitive roots when nitrogen was applied in narrow-rows because thicker roots provide a larger absorption area and help plants absorb more water and nutrients. There was no significant difference in diameter between CR and NCR when nitrogen was applied to wide-rows (**Figure 4F**). Combined with root biomass, root growth showed great spread and adaptability (growth toward nitrogen enrichment) despite the interaction of root systems among plants.

### 3.2 Pot experiment

#### 3.2.1 Yield, biomass allocation, and agronomic characteristics

**Figure 5:**
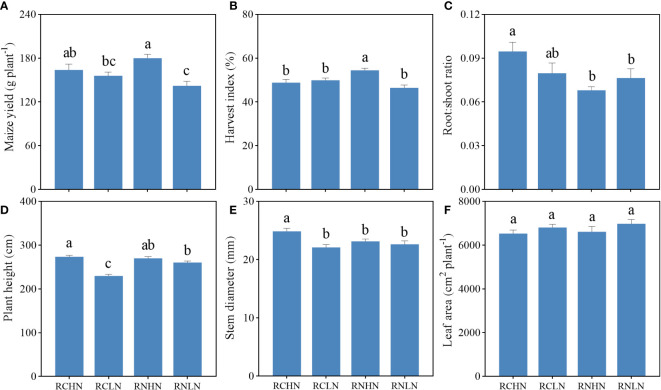
Effects of nitrogen application location and nitrogen level on yield, biomass allocation and agronomic traits in the pot experiment. **(A)** Yield. **(B)** Harvest index. **(C)** Root : shoot ratio. **(D)** Plant height. **(E)** Stem diameter, **(F)** Leaf area index. Different lowercase letters indicate significant difference between treatments (*P* < 0.05).

The yield was basically consistent with the trend of the field experiment and the yield under high nitrogen application was significantly greater than that under low nitrogen application in wide- rows (*F* = 6.53, *P* < 0.01), while the difference of yield in narrow-rows was not significant (**Figure 5A**). The harvest index of the wide-rows with high nitrogen had greater productivity, which was significantly higher than that of the wide-rows with low nitrogen, the narrow-rows with high nitrogen, and narrow-rows with low nitrogen (*F* = 7.20, *P* < 0.01), indicating that the crops could convert light energy into yield more effectively (**Figure 5B**). The root:shoot ratio of high nitrogen in narrow-rows was significantly higher than that in wide-rows (*F* = 3.55, *P* = 0.02), indicating that narrow-rows of roots were more sensitive to competition. There was no significant difference from application in wide-rows, but high nitrogen application in wide-rows was still the lowest (**Figure 5C**). These results indicate that crop biomass allocation was affected by nitrogen application position and nitrogen level, and it may be that nitrogen application position regulates the interaction between crop roots.

The trends in plant height and stem diameter were consistent with those observed in the field experiment (**Figures 5D, E**). Under narrow-row nitrogen application, the plant height and stem diameter were significantly greater in response to high nitrogen application than in response to low nitrogen application (plant height: *F* = 30.97, *P* < 0.01; stem diameter: *F* = 5.88, *P* = 0.001); There was no significant difference from the wide-row nitrogen application. There was no significant difference in leaf area of per plant (**Figure 5F**), but the general trend showed that the leaf area of per plant was higher under low nitrogen application than under high nitrogen application.

#### 3.2.2 Root growth and cultivation behavior

**Figure 6:**
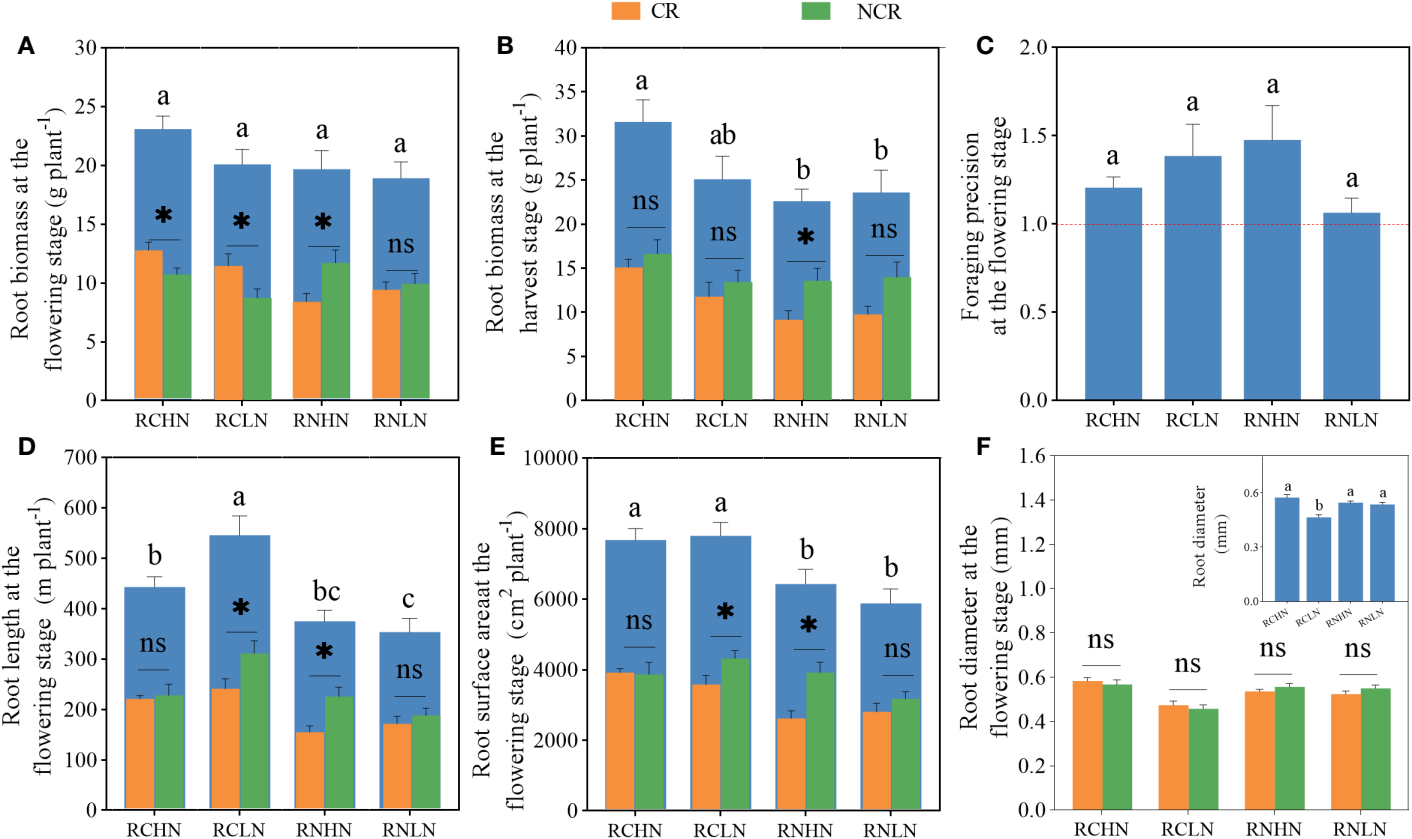
Effects of nitrogen application location and nitrogen level on root traits and foraging behavior in the pot experiment. **(A)** Root biomass at the flowering stage. **(B)** Root biomass at the harvest stage. **(C)** Foraging precision at the flowering stage. **(D)** Root length at the flowering stage. **(E)** Root surface area at the flowering stage. **(F)** Root diameter at the flowering stage; CR: Competitive root, NCR: Non-competiting root. Different lowercase letters indicate significant differences between treatments (*P* < 0.05). ns,* indicates a significant difference between the CR and NCR (*P* > 0.05, *P* < 0.05), respectively.

Unlike the field experiment, there was no significant difference in root biomass in the pot culture (*F* = 1.84, *P* = 0.062), but the CR and NCR biomass exhibited the same trend as those in the field experiment. Except under RNLN, CR and NCR reached a significant levels in the other treatments (RCHN: *F* = 0. 053, *P* = 0.035); RCLN: *F* = 1.49, *P* = 0.048, RNHN: *F* = 1.41, *P* = 0.026; **Figure 6A**). The root biomass in response to narrow-row nitrogen application was greater than that in response to wide-row nitrogen application at maturity and reached a significant level at a high nitrogen level (*F* = 3.069, *F* = 0.044). The biomass was lower in CR than in NCR (**Figure 6B**), indicating that under pot conditions, the application of narrow-row nitrogen promoted root biomass and the CR roots competed more for nitrogen, which was consistent with the accuracy of the root cultivation (**Figure 6C**).

The length (**Figure 6D**) and surface area (**Figure 6E**) of the roots were greater in the narrow- rows than in the wide-rows (root length: *F* = 9.745, *P* < 0.01, surface area: *F* = 6.111, *P* = 0.002), while the effects of nitrogen application position and nitrogen application rate were different between the CR and NCR. Under narrow-rows and low nitrogen conditions, the length and surface area of the NCR roots were significantly greater than those of the CR roots(root length: *F* = 0.888, *P* = 0.045, surface area: *F* = 1.056, *P* = 0.048). Under wide-rows, these traits were significantly greater in the NCR than in the CR (root length: *F* = 0.904, *P* = 0.006, surface area: *F* = 0.674, *P* = 0.003). Furthermore, under narrow-row nitrogen application, the proportion of roots length of different diameters accounted for the total length of root, except for those of 0 to 0.5 mm diameter, was significantly greater than that under low nitrogen application, while the difference in wide-row nitrogen application was mainly between 0.5 to 2.5 mm (**Figure 6F**). The 0 to 2.5 mm root length in RCHN, RCLN, RNHN and RNLN accounted for 96.67, 97.99, 97.38 and 97.20% of the total root length, respectively. Under wide-row nitrogen application, the proportion of root surface area with different diameters was not affected by the nitrogen application rate, while under the narrow- row nitrogen application, low nitrogen significantly affected the root surface area with 0 to 1 mm diameter, and low nitrogen promoted the root surface area with a small diameter to promoted the roots to obtain more nutrients and water in a deeper space. The proportion of the root surface area with 0 to 2.5 mm diameter in RCHN, RCLN, RNHN and RNLN to the total root surface area were 77.01, 83.95, 79.73 and 80.45%, respectively (**Supplementary Tables 3**, **4**). The root diameter of high nitrogen was significantly higher than that of low nitrogen in the application of narrow-row nitrogen application (*F* = 12.566, *P* < 0.01), but the root diameter of CR and NCR were not significantly different under the wide-row nitrogen application. CR was greater than NCR in the narrow-row nitrogen application but was the opposite in response to wide-row nitrogen application, which was consistent with the results of the field experiment. The results indicate that the roots were more sensitive to low nitrogen application under the narrow-rows and that low nitrogen promoted root competition among CR, but this competition decreased with wide-row nitrogen application.

### 3.3 Nitrogen use efficiency

In the field experiment, by comparing different nitrogen levels, the AEN and PFPN were significantly greater at a low nitrogen level than at a high nitrogen level (AEN: *F* = 76.68, *P* < 0.01, PFPN: *F* = 518.15, *P* < 0.01; **Table 1**), indicating that plants generally adjust their physiological and metabolic processes to improve the efficiency of limited nitrogen use and achieve high nitrogen use efficiency under low nitrogen levels. Under a low nitrogen application rate, RC was significantly higher than RN, indicating that nitrogen uptake and utilization by roots could be stimulated by physical contact, chemical signals or shared resources and that the nitrogen uptake capacity of plants would be saturated under a high nitrogen application rate, leading to the inability to use some nitrogen effectively. The above data indicate that plants can limit nitrogen uptake by regulating the root architecture and the nitrogen uptake pathway or may reduce root growth and development and the nitrogen uptake area to avoid the negative impact of nitrogen surplus on plants, which can be confirmed by the root surface area and the root length of CR and NCR. In the pot experiment, under narrow-row nitrogen application, the agronomic efficiency of high nitrogen and the partial productivity of nitrogen were significantly reduced, which was consistent with the field experiment. With wide-row nitrogen application, high nitrogen reduced the partial productivity of nitrogen but improved the agronomic efficiency of nitrogen, and the interaction between the position of N application and the level of N was caused by root reinforcement.

**Table 1:** Effects of nitrogen application location and nitrogen level on nitrogen use efficiency in the field and pot experiments.

Factor		Field Experiment		Pot Experiment	
NAP	NL	AEN (kg kg^-1^)	PFPN (kg kg^-1^)	AEN (kg kg^-1^)	PFPN (kg kg^-1^)
RC	HN	12.39±1.23c	21.76±3.61c	4.73±2.14b	18.21±2.07c
	LN	23.33±3.01a	42.08±5.81a	7.66±1.71a	34.62±1.71a
RN	HN	13.31±1.05c	22.68±2.56c	6.51±1.03a	19.99±2.31c
	LN	17.16±1.69b	35.91±6.33b	4.64±2.46b	31.61±1.09b
NAP (F-value)		0.02	0.01	1.60	0.37
NL (F-value)		76.68**	518.15**	1.18	189.66**
NAP*NL (F-value)		4.41	3.76	24.02**	5.57*

NAP, Nitrogen application position; NL, Nitrogen level; NAP*NL: Interaction effect. * and **indicates significance between treatments (P< 0.05, P < 0.01), respectively. AEN, Agronomic efficiency of N; PFPN, Partial factor productivity of N.

### 3.4 Analysis of key factors of yield and nitrogen use efficiency of CR and NCR in field and pot experiments

**Figure 7:**
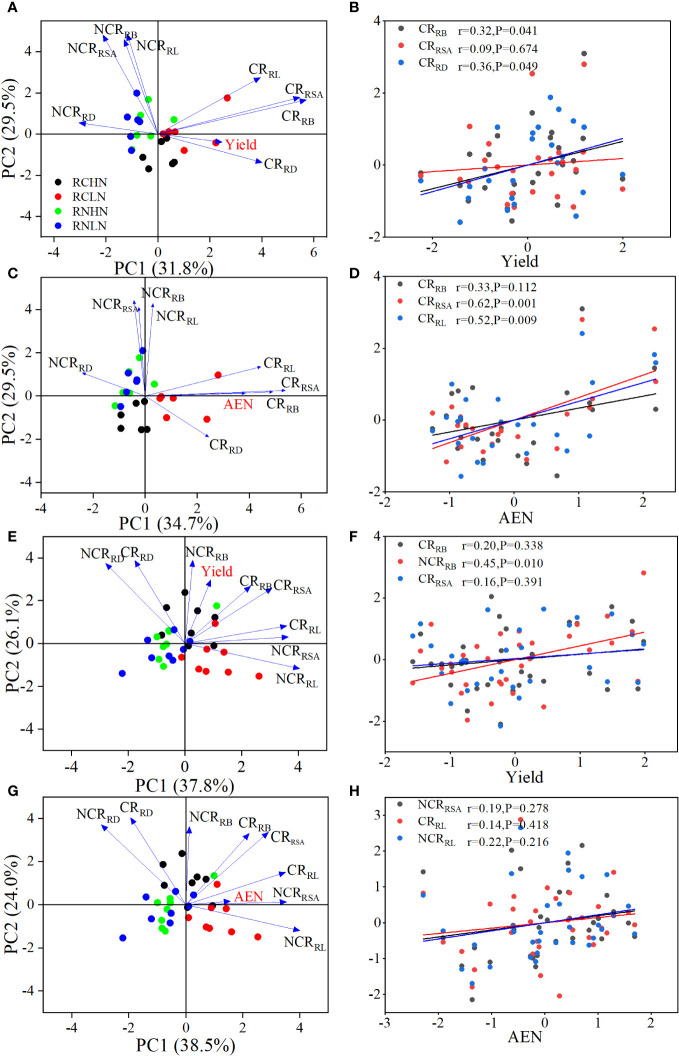
Key factors and correlation analysis of CR and NCR on yield and AEN. **(A)** PCA of yield in the field experiment. **(B)** Correlation analysis of yield in the field experiment. **(C)** PCA of AEN in the field experiment. **(D)** Correlation analysis of AEN in the field experiment. **(E)** PCA of yield in the pot experiment. **(F)** Correlation analysis of yield in the pot experiment. **(G)** PCA of AEN in the pot experiment. **(H)** Correlation analysis of AEN in the pot experiment. CR_RB_: Root biomass of competing root, NCR_RB_: Root biomass of noncompeting root, CR_RSA_: Root surface area of competing root, NCR_RSA_: Root surface area of non-competing roots, CR_RL_: Root length of competing roots, NCR_RL_: Root length of non-competing roots, CR_RD_: Root diameter of competing roots, NCR_RD_: Root diameter of non-competing roots.

According to the PCA results, PC1 and PC2 together explained 61.3% of the yield (**Figure 7A**) and 64.2% of AEN in the field experiment, (**Figure 7C**). The diameter, biomass, and surface area of competitive roots were important factors that affected yield, and the biomass and diameter of competitive roots were significantly positively correlated with yield (**Figure 7B**). The length, surface area, and biomass of competitive roots were important factors affecting the agronomic efficiency of nitrogen fertilizer. The competitive surface area (r = 0.52, *P* = 0.009) and length (r = 0.62, *P* = 0.001) were significantly positively correlated with AEN (**Figure 7D**). In the pot experiment, PC1 and PC2 explained 63.9% of the yield (**Figure 7E**) and 62.5% of the AEN (**Figure 7G**). The biomass and surface area of competitive roots and non-competitive roots were the key factors affecting the yield, and the non-competitive root biomass was positively correlated with the yield (r = 0.45, *P* = 0.01; **Figure 7F**), while the length of competitive roots and the surface area of noncompetitive roots were the key indicators affecting AEN (**Figure 7H**). In summary, the increase in competitive root length, surface area, and biomass were beneficial for yield, while the surface area of non-competitive roots was more strongly correlated with AEN. These findings demonstrate the effects of competitive roots and non-competitive roots on yield and AEN on different nitrogen application positions.

## 4 Discussion

### 4.1 Importance of the position of nitrogen application to root seeking behavior and productivity

In agroecosystems, root length, root surface area, root biomass, and other indicators are widely considered important factors that determine nitrogen absorption and utilization in the soil by crops. With the continuous development of intensive and diversified planting, the interactions between roots have become increasingly complex (Ma et al., 2022). In wide-and narrow-row cropping systems, changing the position of nitrogen application is particularly prominent in regulating plant growth and root distribution patterns, affecting the dynamic changes and competitive relationships of the plant population (Li et al., 2021). In this study, field experiments showed that, in a wide- and narrow-row planting system, the application of wide-row nitrogen had obvious positive effects on increasing total root biomass, total root surface area, and root length compared to those in narrow-row planting systems, especially under high nitrogen conditions (**Figures 4A, D, E**). This may be due to the excessive absorption of a small amount of nutrient resources by two adjacent maize roots in narrow-rows, which reduces the concentration of nutrients, thus causing the competition of root with each other, avoiding the space and nutrients occupied by adjacent plant roots, and showing asymmetric growth in space for adjacent plant roots (i.e., the growth of noncompetitive roots is better than that of competitive roots) (Kroon et al., 2009; Wang et al., 2016; Zhang et al., 2022). The application of nitrogen in a wide-row is equivalent to a ‘resource pull’, which promotes root growth on the non-competitive side, and this ‘resource pull’ becomes larger with increasing nitrogen application rate (Kembel and Cahill, 2005), increasing the root surface area and length ratio of 0 to2.5 mm in diameter to obtain more nutrients (**Supplementary Tables 1**, **2**).

However, in narrow-row nitrogen application, although it is generally believed that nitrogen is more concentrated in the soil (compared to wide-row nitrogen application, the degree of heterogeneous soil resources is smaller), it is easier for roots to actively forage resources (roots consume less energy in the process of foraging resources, and roots are easier to proliferate in large quantities between narrow-rows). From this perspective, the application of narrow-row nitrogen is beneficial to plant growth. However, this kind of root proliferation weakens the ability of non-competitive roots to actively forage resources (reducing the biomass allocation of non-competitive roots). Furthermore, root competition for nitrogen between narrow-rows shows that plants generally increase their investment in roots, according to the theory of ‘competition-induced root growth’ (Chen et al., 2021; Pretzsch, 2022), and for plants, root growth tends to occupy the growth resources (space and nutrients) of neighboring plants rather than increase the cost of investing in themselves (Ljubotina and Cahill, 2019; Cabal, 2022). The nutrient acquisition capacity of roots has become a limiting factor for growth. Additionally, from the discovery of the proportion of root length and surface area of 0 to 2.5 mm (**Supplementary Tables 3**, **4**), under narrow-row nitrogen application, the proportion of fine roots was less than in wide-row nitrogen application, which is more unfavorable for plants nutrients absorption. From this perspective, this easy foraging process between narrow-rows is unfavorable for plant growth. Will plants have this beneficial and unfavorable effect under narrow-row nitrogen application? This may depend on which roots between two adjacent plants has foraging ability first, thus occupying a favorable niche because the competitive effect of individuals has a certain correlation with the root foraging scale (root biomass) and the root foraging rate (time required to reach the patch) (Dell et al., 2014). Generally, the interaction between the heterogeneous nitrogen supplication and the existence of adjacent plants increased the input of maize foraging resources into nitrogen-rich areas.

The nitrogen availability is also an important factor that affects the root cultivation process. Previous studies have also shown that only when the nitrogen concentration in the soil is at a low level can the root system fully exploit the biological potential of ‘fertilizer displacement’, and this effect is obviously inhibited under conditions of excessive nitrogen supplication (Broadbent et al., 2018; Dybzinski et al., 2019). This was consistent with the results of narrow-row nitrogen application in a field experiment, but the effect was the opposite under the wide-row nitrogen application. Therefore, when there is root interaction, changing the nitrogen application position can adjust root growth and change root distribution to improve overall root-seeking behavior. In addition, unlike the field experiment, the process and behavior of root cultivation in pot culture differ. Under narrow-row nitrogen application, the length and surface area of competitive roots and non-competitive roots were opposite those in the field experiment, while those under wide-row nitrogen application were consistent. It may be that the space resources occupied by competitive roots in potted plants are smaller than those in large fields, and the volume of potted plants (especially the nutrient pots where competitive roots are located) may limit the growth of plant roots and strengthen contact between plants (**Figure 2C**), and there is no exchange of nutrients (Chen et al., 2012).

Increased foraging precision usually the performance of the adaptability of the plant to environmental resources, which is helpful for the absorption of plant nutrients and enhances the competitive advantage of the plant. However, in field studies, although the precision of finding nutrients during narrow-row nitrogen application was improved compared to wide-row nitrogen application (at the same nitrogen level), the comparative yield of maize did not increase, especially in the case of greater nitrogen application (**Figures 3A, C**). According to the “competitor–stress-tolerant–disturbance-tolerant” theory (Grime, 1974), in an environment with high productivity (under high nitrogen conditions), the intensity of competition among plants will increase because the biomass of adjacent plants aboveground is positively linearly correlated with the intensity of competition, and biomass of plant increases with the improvement of soil fertility. However, the further expansion of the root absorption area, the fiercer the competition in fertile soil. This may be an important reason why the yield under wide-row nitrogen application is significantly higher than that of a narrow-row nitrogen application under a high nitrogen application rate (Zhai et al., 2017; Monson et al., 2022). Furthermore, when the nutrient patch is in the middle of two maize plants, many roots grow coincidently in the nutrient patch area greatly improving the intensity of competition between plants. However, when nitrogen is applied in wide-rows, non-competitive roots show a pattern of mutual avoidance distribution in biomass, surface area, and length compared to the competitive roots, reducing the competition of roots for common resources. Our results have been verified in related studies, involving woody, grassland, and food crops (Cahill et al., 2010; Lorts and Lasky, 2020; Shen et al., 2020).

### 4.2 Relationship between root seeking behavior and nitrogen use efficiency

The spatial distribution of plant roots directly determines their uptake and utilization of nutrients and ultimately manifests itself in the uptake of aboveground nutrients and the accumulation of biomass. In the field experiment, the AEN and PFPN under wide-row nitrogen application were greater than those under narrow-row nitrogen application under a high nitrogen application rate, while that of the narrow-row nitrogen application were significantly greater than that of the wide-row nitrogen application under low nitrogen, which indicated that the spatial heterogeneity of nutrients could have specificity for the absorption of shoot nutrients based on the nitrogen position. The reason may be that although narrow-row nitrogen application can promote root proliferations in patches under high nitrogen application. Root proliferation is related to its type. Generally, we think that roots with a small diameter (0 to 2.5 mm) have a greater absorption capacity (Jackson, 2000). According to **Figure 4F**, the diameter of the competitive roots was significantly higher than that of the non-competitive roots under nitrogen application in narrow-row. Although a larger root diameter has a larger root surface area, this root proliferation is not necessarily beneficial in absorbing more nutrients. Stimulation of root proliferation in nutrient-rich areas may not be the only reason for improving plant ‘income’. According to the hypothesis of ‘ineffective proliferation’, there may be a trade-off between long-lived coarse absorber roots and fast-foraging fine absorber roots (Hodge et al., 2010; Mommer et al., 2012; Zhu et al., 2022).

Most studies have shown that during competition, the amount of nutrients obtained by plants is positively related to the size of their roots, which can improve the efficiency of nutrient utilization of plants in nutrient patches in a short time and have a positive promotional effect on the growth of above-ground parts (Jansen et al., 2006; Jing et al., 2012). However, in the field experiments, nutrients can be consumed rapidly and flow through the nature rainfall, making this advantage last for a short time. Eventually, fine roots with a short life expectancy will cause a large amount of energy loss. Additionally, the reason for the low nitrogen utilization rate of narrow-row nitrogen application under high nitrogen may be that when there are nutrient patches, the different times and degrees of utilization of two maize roots contacting the nutrient patches do not lead to a linear correlation between nutrient absorption by the roots and root size, demonstrating that heterogeneous nutrients lead to asymmetric competition between two maize roots. Plants that arrive first at the local supply of nutrients will occupy this part of the nutrient patch, ‘neutralizing’ nitrogen utilization efficiency between the two maize plants (Raynaud and Leadley, 2005; Weiner and Damgaard, 2006; Rasmussen et al., 2019). Obviously, the local supply of nutrients has different effects on root behavior and root foraging. However, in addition to nutrient attributes, the sensitivity of plant roots to nutrients, the length of the plant growth cycle, the occurrence of interactions between roots, and the occurrence of interactions between competition and reciprocity strongly influence plant nutrient absorption and root plasticity. In the future, the ecological functions of nutrient patches in root foraging behavior should be systematically evaluated on different time scales to explain the quantitative regulation of root foraging behavior.

### 4.3 Regulation of nitrogen application position on above-ground and underground parts

The allocation between the roots and crowns of plants is determined by the minimum access to resources and balanced growth among adjacent roots (Yuan et al., 2021). In wide-row nitrogen applications, the sharing of resources among roots is more balanced than that in narrow-row nitrogen applications. The growth environment of whole plant is mild, while competition among above-ground, underground, and whole plants is weak (Venterink and Güsewell, 2010). Therefore, in this case, neighboring plants in the underground part will experience less competition than other plants, and plants may change from underground to above-ground competition for light resources when weighing the competition relationship. However, the overall intensity of competition does not change with the change in productivity. Therefore, plants increase their height and leaf area, to improve the productivity of the upper part, and to reduce the root:shoot ratio. During narrow-row nitrogen application, for two maize plants, and the resource supply is concentrated, the interaction between plants and roots increase the competition between the aboveground and underground, and the total effect of competition increases. According to the hypothesis of balanced growth of roots and shoots, plant growth is limited by constant light resources, and the competition between soil resources and roots decreases with increasing resource availability, which may be a potential mechanism to explain the spatial heterogeneity of resources and the coordination of biomass allocation by root competition (Meziane, 2010; Poorter and Sack, 2012). In other words, the response of plants to the spatial heterogeneity of soil nutrients and root interactions and the mechanism of searching for nutrients has become one of the hot spots in ecological research. Clarifying its mechanism can not only improve our understanding of plant ecosystems, but also help optimize the application of farmland planting, ecological restoration, and plant protection.

## 5 Conclusions

Field and pot experiments showed that narrow-and wide-row nitrogen applications improved the precision of foraging precision. Wide-row nitrogen application reduced the sensitivity of underground roots to soil resources, improved the overall productivity of the crops, optimized the investment ratio of the crops to the above-ground and underground parts, and reduced the efficiency of nitrogen use under low nitrogen application. To better understand the relationships among resource availability, root interactions, and nitrogen spatial heterogeneity, it is necessary to clarify the influence of aboveground light resources on this process. Specifically, it is necessary to clarify whether aboveground resources coordinate root interactions and nitrogen spatial heterogeneity in this process. Therefore, to improve the sustainability of crop production and promote healthy soil development, this process deserves further study.

## Data availability statement

The original contributions presented in the study are included in the article/**Supplementary Material**. Further inquiries can be directed to the corresponding authors.

## Author contributions

SZ: Data curation, Investigation, Validation, Writing – original draft. PX: Data curation, Investigation, Methodology, Writing – original draft. JC: Data curation, Investigation, Methodology, Writing – original draft. QX: Data curation, Methodology, Writing – original draft. GL: Investigation, Writing – original draft. JT: Data curation, Investigation, Writing – original draft. BW: Conceptualization, Funding acquisition, Project administration, Supervision, Writing – review & editing. FZ: Conceptualization, Funding acquisition, Project administration, Supervision, Visualization, Writing – review & editing.
