# Supplementary material for: Optimizing nitrogen application position to change root distribution in soil and regulate maize growth and yield formation in a wide–narrow row cropping system: pot and field experiments
Source: Front Plant Sci. 2024 Jan 24;15:1298249. doi: 10.3389/fpls.2024.1298249 (PMC10847348; doi:10.3389/fpls.2024.1298249)
Supplement: Supplementary file 1 [file Table_1.docx]

**Table 1 Percentage (%) of root length with different diameters of total length in the field experiment**

| Treatments | 0<.L.<= 0.5000000 | 0.5000000 <.L.<= 1.0000000 | 1.0000000 <.L.<= 1.5000000 | 1.5000000 <.L.<= 2.0000000 | 2.0000000 <.L.<= 2.5000000 | 2.5000000 <.L.<= 3.0000000 | 3.0000000 <.L.<= 3.5000000 | 3.5000000 <.L.<= 4.0000000 | 4.0000000 <.L.<= 4.5000000 | .L.>4.5000000 |
| --- | --- | --- | --- | --- | --- | --- | --- | --- | --- | --- |
| RCHN | 72.01±1.50b | 15.69±0.60a | 5.65±0.35ab | 2.56±0.22a | 1.54±0.14a | 0.92±0.07a | 0.51±0.05a | 0.34±0.03a | 0.24±0.03a | 0.49±0.08a |
| RCLN | 72.32±0.67ab | 15.54±0.45ab | 5.68±0.19a | 2.55±0.10a | 1.50±0.06a | 0.88±0.04ab | 0.48±0.02a | 0.33±0.02ab | 0.23±0.01a | 0.45±0.05a |
| RNHN | 73.21±1.81ab | 15.17±0.67ab | 5.42±0.38ab | 2.44±0.24a | 1.43±0.19a | 0.82±0.13ab | 0.45±0.07a | 0.31±0.05ab | 0.22±0.04a | 0.47±0.09a |
| RNLN | 74.34±1.59a | 14.77±0.56b | 5.18±0.39b | 2.31±0.24a | 1.35±0.17a | 0.77±0.08b | 0.43±0.06a | 0.27±0.05b | 0.18±0.03b | 0.35±0.05b |
| *F* | 2.55 | 2.52 | 2.25 | 1.44 | 1.53 | 2.34 | 1.72 | 2.32 | 3.84 | 3.7 |
| *P* | 0.08 | 0.08 | 0.11 | 0.25 | 0.23 | 0.10 | 0.19 | 0.10 | 0.02 | 0.02 |

**Note:** 0<.L.<= 0.5000000 indicates the root length between 0 and 0.5000000mm in diameter. RCHN: narrow row with high nitrogen, RCLN: narrow row with low nitrogen, RNHN: wide row with high nitrogen, RNLN: wide row with low nitrogen. Significance of the same column with different lowercase alphabets at *P* < 0.05.

**Table 2 Percentage（%） of surface area of different root diameters to total root surface area in the field experiment**

|  | 0 <.SA.<= 0.5000000 | 0.5000000 <.SA.<= 1.0000000 | 1.0000000 <.SA.<= 1.5000000 | 1.5000000 <.SA.<= 2.0000000 | 2.0000000 <.SA.<= 2.5000000 | 2.5000000 <.SA.<= 3.0000000 | 3.0000000 <.SA.<= 3.5000000 | 3.5000000 <.SA.<= 4.0000000 | 4.0000000 <.SA.<= 4.5000000 | .SA.> 4.5000000 |
| --- | --- | --- | --- | --- | --- | --- | --- | --- | --- | --- |
| RCHN | 29.32±1.6a | 22.19±0.54a | 13.88±0.30a | 8.84±0.37a | 6.86±0.36a | 5.04±0.19a | 3.31±0.20a | 2.59±0.14a | 2.1±0.20a | 5.88±0.84a |
| RCLN | 29.56±0.72a | 22.37±0.62a | 14.21±0.26a | 8.97±0.24a | 6.79±0.24a | 4.9±0.20a | 3.17±0.15a | 2.52±0.16a | 2.04±0.15a | 5.48±0.74ab |
| RNHN | 30.54±2.24a | 22.24±0.60a | 13.81±0.30a | 8.76±0.35a | 6.57±0.53a | 4.67±0.48a | 3.04±0.37a | 2.45±0.31a | 1.98±0.25a | 5.96±1.05a |
| RNLN | 32.11±2.11a | 22.72±0.56a | 13.88±0.4a | 8.71±0.47a | 6.54±0.50a | 4.61±0.27a | 3.03±0.30a | 2.25±0.32a | 1.67±0.25b | 4.49±0.44b |
| *F* | 2.56 | 0.84 | 1.65 | 0.48 | 0.71 | 2.08 | 1.18 | 1.81 | 3.93 | 3.58 |
| *P* | 0.08 | 0.48 | 0.20 | 0.69 | 0.55 | 0.13 | 0.34 | 0.17 | 0.02 | 0.03 |

Note: 0<.SA.<= 0.5000000 indicates the root surface area between 0 and 0.5000000mm in diameter. RCHN: narrow row with high nitrogen, RCLN: narrow row with low nitrogen, RNHN: wide row with high nitrogen, RNLN: wide row with low nitrogen. Significance of the same column with different lowercase alphabets at *P* < 0.05.

**Table 3 Percentage (%) of root length with different diameters of total length in the pot experiment**

|  | 0<.L.<= 0.5000000 | 0.5000000 <.L.<= 1.0000000 | 1.0000000 <.L.<= 1.5000000 | 1.5000000 <.L.<= 2.0000000 | 2.0000000 <.L.<= 2.5000000 | 2.5000000 <.L.<= 3.0000000 | 3.0000000 <.L.<= 3.5000000 | 3.5000000 <.L.<= 4.0000000 | 4.0000000 <.L.<= 4.5000000 | .L.>4.5000000 |
| --- | --- | --- | --- | --- | --- | --- | --- | --- | --- | --- |
| INHN | 69.31±0.99b | 16.15±0.62b | 6.32±0.33b | 3.02±0.18b | 1.87±0.09a | 1.15±0.07a | 0.66±0.06a | 0.46±0.06a | 0.33±0.06a | 0.72±0.25a |
| INLN | 74.16±2.54a | 14.91±1.07c | 5.25±0.68c | 2.34±0.35c | 1.33±0.21b | 0.76±0.12b | 0.41±0.07c | 0.28±0.05c | 0.19±0.04c | 0.37±0.10b |
| NIHN | 60.07±1.48c | 25.00±1.51a | 7.42±0.34a | 3.20±0.23a | 1.69±0.19a | 1.02±0.12a | 0.59±0.09ab | 0.37±0.05b | 0.26±0.04b | 0.37±0.07b |
| NILN | 69.61±1.78b | 16.58±0.60b | 6.32±0.46b | 2.95±0.28b | 1.74±0.18a | 1.02±0.13a | 0.57±0.08b | 0.40±0.06b | 0.27±0.05b | 0.54±0.15b |
| *F* | 76.76 | 142.62 | 24.15 | 13.82 | 12.33 | 14.55 | 13.64 | 12.1 | 9.79 | 8.27 |
| *P* | ＜0.01 | ＜0.01 | ＜0.01 | ＜0.01 | ＜0.01 | ＜0.01 | ＜0.01 | ＜0.01 | ＜0.01 | ＜0.01 |

**Note:** 0<.L.<= 0.5000000 indicates the root length between 0 and 0.5000000mm in diameter. RCHN: narrow row with high nitrogen, RCLN: narrow row with low nitrogen, RNHN: wide row with high nitrogen, RNLN: wide row with low nitrogen. Significance of the same column with different lowercase alphabets at *P* < 0.05.

**Table 4 Percentage （%）of surface area of different root diameters to total root surface area in the pot experiment**

|  | 0<.SA.<= 0.5000000 | 0.500000 0<.SA.<= 1.0000000 | 1.0000000 <.SA.<= 1.5000000 | 1.5000000 <.SA.<= 2.0000000 | 2.0000000 <.SA.<= 2.5000000 | 2.5000000 <.SA.<= 3.0000000 | 3.0000000 <.SA.<= 3.5000000 | 3.5000000 <.SA.<= 4.0000000 | 4.0000000 <.SA.<= 4.5000000 | .SA.> 4.5000000 |
| --- | --- | --- | --- | --- | --- | --- | --- | --- | --- | --- |
| INHN | 25.27±1.06b | 20.72±1.2b | 14.06±0.93a | 9.45±0.63a | 7.51±0.31a | 5.67±0.16a | 3.86±0.26a | 3.11±0.32a | 2.51±0.41a | 7.84±2.65a |
| INLN | 32.06±3.07a | 22.84±0.8a | 13.96±0.94a | 8.72±0.72b | 6.37±0.58b | 4.48±0.42c | 2.89±0.31c | 2.25±0.30c | 1.72±0.26c | 4.70±1.25b |
| NIHN | 25.82±1.74b | 21.86±0.76a | 14.80±0.26a | 9.82±0.33a | 7.43±0.49a | 5.48±0.43ab | 3.67±0.37ab | 2.92±0.27ab | 2.27±0.24ab | 5.94±0.61ab |
| NILN | 26.98±1.88b | 22.1±0.97a | 14.58±0.54a | 9.55±0.41a | 7.24±0.34a | 5.22±0.38b | 3.45±0.27b | 2.76±0.29b | 2.13±0.26b | 5.99±1.49ab |
| *F* | 15.70 | 6.00 | 2.16 | 5.18 | 9.68 | 14.52 | 13.03 | 10.84 | 8.47 | 4.21 |
| *P* | ＜0.01 | 0.003 | 0.15 | 0.006 | ＜0.01 | ＜0.01 | ＜0.01 | ＜0.01 | ＜0.01 | 0.014 |

Note: 0<.SA.<= 0.5000000 indicates the root surface area between 0 and 0.5000000mm in diameter. RCHN: narrow row with high nitrogen, RCLN: narrow row with low nitrogen, RNHN: wide row with high nitrogen, RNLN: wide row with low nitrogen. Significance of the same column with different lowercase alphabets at *P* < 0.05.
